# Impact of the COVID‐19 pandemic on urological cancers: The surgical experience of two cancer hubs in London and Milan

**DOI:** 10.1002/bco2.135

**Published:** 2022-01-27

**Authors:** Maria J. Monroy‐Iglesias, Sonpreet Rai, Francesco A. Mistretta, Graham Roberts, Harvey Dickinson, Beth Russell, Charlotte Moss, Rita De Berardinis, Matteo Ferro, Gennaro Musi, Christian Brown, Rajesh Nair, Ramesh Thurairaja, Archana Fernando, Paul Cathcart, Azhar Khan, Prokar Dasgupta, Sachin Malde, Marios Hadijpavlou, Saoirse Dolly, Kate Haire, Marta Tagliabue, Ottavio de Cobelli, Ben Challacombe, Mieke Van Hemelrijck

**Affiliations:** ^1^ Faculty of Life Sciences and Medicine, Translational Oncology & Urology Research (TOUR) King's College London London UK; ^2^ Department of Urology Guy's and St Thomas' NHS Foundation Trust London UK; ^3^ Division of Urology European Institute of Oncology IRCCS Milan Italy; ^4^ South East London Cancer Alliance London UK; ^5^ Division of Otolaryngology and Head and Neck Surgery European Institute of Oncology IRCCS Milan Italy; ^6^ Department of Oncology and Haemato‐oncology University of Milan Milan Italy; ^7^ Department of Medical Oncology Guy's and St Thomas' NHS Foundation Trust London UK; ^8^ Department of Biomedical Sciences University of Sassari Sassari Italy

**Keywords:** cancer, COVID‐19, epidemiology, surgery, urology, uro‐oncology

## Abstract

**Objective:**

To report on the outcomes of urological cancer patients undergoing radical surgery between March–September 2020 (compared with 2019) in the European Institute of Oncology (IEO) in Milan and the South East London Cancer Alliance (SELCA).

**Materials and Methods:**

Since March 2020, both institutions implemented a COVID‐19 minimal ‘green’ pathway, whereby patients were required to isolate for 14 days prior to admission and report a negative COVID‐19 polymerase chain reaction (PCR) test within 3 days of surgery. COVID‐19 positive patients had surgery deferred until a negative swab. Surgical outcomes assessed were: American Society of Anaesthesiologists (ASA) grade; surgery time; theatre time; intensive care unit (ICU) stay >24 h; pneumonia; length of stay (LOS); re‐admission. Postoperative COVID‐19 infection rates and associated mortality were also recorded.

**Results:**

At IEO, uro‐oncological surgery increased by 4%, as compared with the same period in 2019 (*n* = 515 vs. 534). The main increase was observed for renal (16%, *n* = 98 vs. 114), bladder (24%, *n* = 45 vs. 56) and testicular (27%, *n* = 26 vs. 33). Patient demographics were all comparable between 2019 and 2020. Only one bladder cancer patient developed COVID‐19, reporting mild/moderate disease. There was no COVID‐19 associated mortality. In the SELCA cohort, uro‐oncological surgery declined by 23% (*n* = 403 vs. 312) compared with the previous year. The biggest decrease was seen for prostate (−42%, *n* = 156 vs. 91), penile (−100%, *n* = 4 vs. 0) and testicular cancers (−46%, *n* = 35 vs. 24). Various patient demographic characteristics were notably different when comparing 2020 versus 2019. This likely reflects the clinical decision of deferring COVID‐19 vulnerable patients. One patient developed COVID‐19, with no COVID‐19 related mortality.

**Conclusion:**

The COVID‐19 minimal ‘green’ pathways that were put in place have shown to be safe for uro‐oncological patients requiring radical surgery. There were limited complications, almost no peri‐operative COVID‐19 infection and no COVID‐19‐related mortality in either cohort.

## 1 INTRODUCTION

The new severe acute respiratory syndrome SARS‐CoV‐2 (COVID‐19) has had a profound global impact on urological patients, with a reported genitourinary cancer surgery deferral rate of up to 53% globally.^1^ The United Kingdom (UK) and Italy are two of the most affected countries in Europe. After a general lockdown began in both countries to avoid the spread of infection, National Health Service England (NHSE) and the Italian National Health System (INHS) interrupted all non‐urgent surgery, outpatient clinics and rehabilitation services.^2^, ^3^ Hospitals reorganised services and redeployed staff to prioritise the management of COVID‐19 patients.^4^, ^5^ Although it had not yet been established, uro‐oncological surgical care was being postponed on the assumption that COVID‐19 infection was associated with higher postoperative morbidity and mortality.^6^ An early international multicentre study looking at perioperative COVID‐19 infection reported a postoperative 30‐day mortality of 24%, with a pulmonary complication rate of 50% in patients with perioperative COVID‐19.^7^ Routine outpatient clinics and elective surgery was cancelled to allocate resources, and only urgent and high‐risk cancer surgery was performed.^4^ Over a year into the COVID‐19 pandemic, epidemiological evidence has now reported a significant decrease in urgent cancer referrals.^8^ This has led to a delay in cancer diagnoses and treatment resulting in more cases of advanced disease at referral.^5^ A recent cohort study reported that substantial increases in the number of cancer deaths in the UK are to be expected as a result of diagnostic delays due to COVID‐19.^5^ In this context, safe pathways and guidelines were developed to minimise the risk of contracting COVID‐19 while balancing treatment options.^9^ A similar recent study looking at overall surgical practice in cancer patients in the same two populations reported favourable outcomes when implementing these pathways.^10^

The European Institute of Oncology, IRCCS (IEO) in Milan is one of the largest cancer hospitals in Italy. The South East London Cancer Alliance (SELCA) includes three major teaching hospital trusts; Guy's and St. Thomas' NHS Foundation Trust, Lewisham and Greenwich NHS Trust, and Kings College Hospital NHS Foundation Trust. London and Milan were both at the epicentre of the first COVID‐19 wave and were forced to implement strict new pathways to provide cancer care. The main objective of this study was to assess the safety and reliability of the COVID‐19 minimal ‘green’ pathways implemented for urological cancer patients receiving radical uro‐oncological surgery in both these centres. We aim to describe these pathways and report on the demographic characteristics and surgical outcomes for urological patients undergoing surgery. The aim is to inform other cancer centres performing urological surgery and help organise their COVID‐19 pathways.

## 2 METHODS

### 2.1 Study population

The study population consisted of all patients undergoing radical surgery with curative intent for urological cancers. In the IEO, all patients undergoing urological surgery between 1 March and 30 September 2020, as well as the comparable period in 2019 were included. In the SELCA group, all patients undergoing radical uro‐oncological surgery between 23 March and 8 September 2020, and the comparable period in 2019 were included.

Data collected from all participants included gender, age, socioeconomic status (SES), ethnicity, comorbidities (hypertension, diabetes mellitus [DM], respiratory disease, renal impairment, liver disease, cardiovascular disease [CVD]), performance status (according to the world health organisation [WHO]),^11^ body mass index (BMI), tumour site, American Society of Anaesthesiologists (ASA) classification,^12^ surgery time, theatre time, >24 h of intensive care unit (ICU) stay, length of stay (LOS), readmissions, complications according to the Clavien–Dindo classification,^13^ postoperative COVID‐19 status, and death by any cause. Data on COVID‐19 severity (mild/moderate/severe) and COVID‐19 related deaths were only available for the IEO study population. Mild/moderate disease was defined as having the various signs and symptoms of COVID‐19 (i.e., fever, cough, flu‐like symptoms and anosmia) and oxygen saturation (SpO_2_) ≥94% on room air; while severe disease was defined as SpO_2_ <94% on room air, a ratio of arterial partial pressure of oxygen to fraction inspired oxygen (PaO_2_/FiO_2_) <300 mmHg, respiratory frequency >30 breaths/min, or lung infiltrates >50%.^14^ Weekly number of COVID‐19 cases in London were extracted from the Public Health England Coronavirus dashboard.^15^ Weekly number of COVID‐19 cases in Milan were extracted from the Italian Ministry of Health portal.^16^, ^17^

### 2.2 Patient pathways

#### 2.2.1 IEO

From the outset of the pandemic, doctors and nursing medical staff took on the responsibility of assessing for the absence of signs or symptoms of COVID‐19 and managing the urgency and priority of outpatient visits. Only patients themselves could enter the hospitals with no visitors permitted. The use of surgical masks was compulsory, and body temperature was measured by infrared thermometers, where patients with a body temperature <37.5°C were permitted to enter the hospital. Before elective admission, all patients had a telephone triage to assess their current health status, lack of COVID‐19 symptoms (fever, cough, flu‐like symptoms and anosmia) and possible contact with COVID‐19 positive people or those with symptoms indicative of COVID‐19. From 1 September 2020, a nasopharyngeal swab for COVID‐19 was collected for all urological patients at the surgical pre‐ assessment visit. With COVID‐19 negative patients, surgery was scheduled within 3 to 5 days from the swab. In COVID‐19 positive patients, two consecutive negative swabs and 14 days of self‐isolation was required to perform surgery.

**FIGURE 1:**
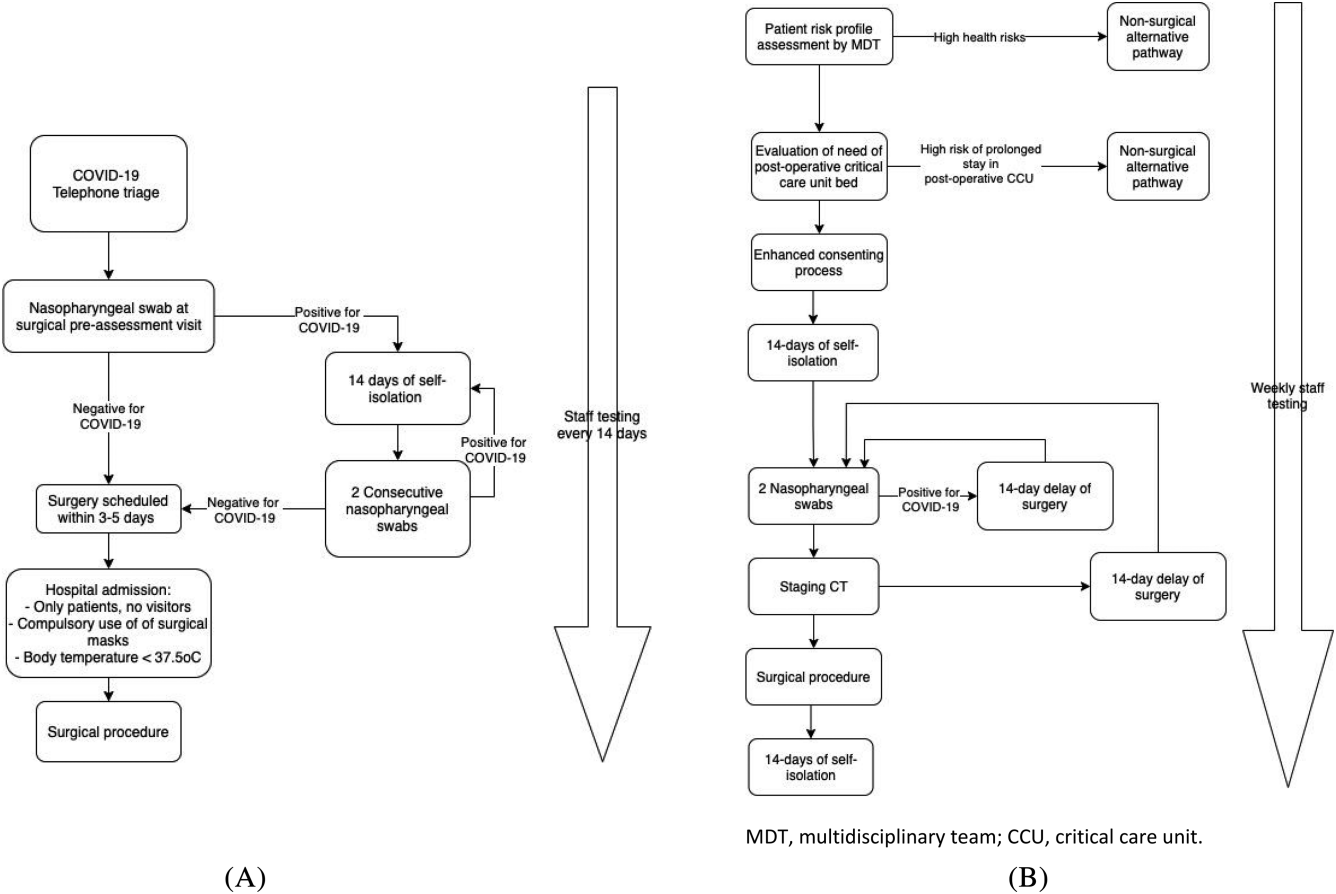
(A) Flow chart of pre‐operative COVID‐19 ‘green’ pathway followed by the European Institute of Oncology (IEO). (B) Flow chart of pre‐operative COVID‐19 ‘green’ pathway followed by South East London Cancer Alliance (SELCA)

An anaesthetic protocol was devised to minimise aerosol generation and potential exposure to undetected COVID‐19 infection in patients with false negative swab results. All involved staff were required to wear full personal protection equipment (PPE) and only the anaesthetist and operating department practitioner (ODP) had access to the operating theatre during the patient's anaesthetic intubation. During surgery, all staff involved had to wear full PPE throughout the procedure. In the postoperative period, patients were in single rooms with surgical masks and all visiting healthcare professionals were required to donn full PPE when entering the room. Health personnel were also swabbed every 15 days to detect asymptomatic vectors. Figure 1A illustrates the pre‐operative patient and staff COVID‐19 protocols for the IEO. The IEO data collection was approved by the European Institute of Oncology Ethics Committee (code. IEO 2432).

#### 2.2.2 SELCA

A multidisciplinary team, formed by a panel of clinicians and a dedicated tumour board, assessed patients' risk profiles according to new UK government guidance in relation to their co‐morbidities and the potential negative effects of COVID‐19. If the health risks were deemed too high, patient care was directed to an alternative non‐surgical pathway. The need for a postoperative critical care unit (CCU) bed was evaluated and if deemed too high risk or prolonged stay was expected (i.e., performance status of >3), alternative treatments were considered. An enhanced consenting process was utilised, which included agreed levels of care in the postoperative period with some patients electing not to have CCU care if their condition deteriorated after surgery. All patients were instructed to self‐isolate for 14 days to minimise the risk of acquiring COVID‐19 infection in the peri‐operative period. Two negative swabs were required during surgical pre‐assessment in order to proceed to surgery. If the staging computed tomography (CT) scan of the chest identified incidental COVID‐19 disease, surgery would be delayed for at least 14 days, irrespective of whether patients had the required two negative swabs.

At the time of anaesthetic induction, all patients were intubated in the operating theatre with only the anaesthetic team within the operating theatre suite and wearing full PPE. Once the endotracheal tube was placed, a wait time of 20 min was mandatory before entering the theatre suite; this was to allow for adequate air exchanges to occur and minimise the exposure to any aerosol generated during intubation.

During surgery itself, only essential personnel were in the operating theatre and full PPE was worn by all theatre staff. There was no teaching or training in this period. All surgical procedures were consultant led and delivered to optimise procedural efficiency and utilisation of theatre time.

To optimise sterility of surgical wounds, povidone‐iodine (Betadine) preparation was used for all surgery site cleaning and preparation. A smoke and gas insufflation and evacuation system (ConMed Airseal®) was used in all minimally invasive aerosol generating cases. Intra‐abdominal pressure was limited to 12 mmHg with lower insufflation pressures used as standard when possible. Robotic/laparoscopic ports were never vented to the atmosphere. A retrieval bag was chosen that could be placed through a sealed port. At the end of the procedure all gas was aspirated, via a filtered suction unit, from the least dependent port.

Once surgery was complete, the patient remained in the theatre for a further 20 min following extubation, prior to being transferred to the recovery room. Additionally, there was a mandatory simulation training programme for all theatre staff, which included putting on (‘donning’) and removing (‘doffing’) of PPE techniques, intubation techniques and failed intubation drills.

Full PPE used by all physicians in both Institutes comprised of: filtering face piece 2 (FFP2) mask, in addition to a surgical mask, water‐repellent disposable gown, double gloves, and protective goggles or visor. In the surgical theatres the protocol was to have an area of donning and doffing in line with Public Health England (PHE) guidelines.^18^, ^19^ Lastly, health personnel were swabbed every 7 days to detect asymptomatic vectors. Figure 1B illustrates the pre‐operative patient and staff COVID‐19 protocols for SELCA. The data collection for SELCA was approved by and Guy's Cancer Cohort (Reference number: 18/NW/0297).

### 2.3 Statistical analyses

Descriptive statistics were performed to describe baseline socio‐demographic and clinical characteristics, surgical and COVID‐19 outcomes. Absolute and relative frequencies for categorical variables, median values and interquartile ranges (IQRs) for continuous variables are reported. Differences in patient characteristics between 23 March and 8 September 2020 and the comparable period in 2019 were evaluated with the *Z*‐score test for two population proportions.

## 3 RESULTS

At the IEO, there were 534 uro‐oncological surgical procedures with curative intent performed from March to September 2020 (303 prostate cancer, 56 bladder cancer, 114 renal cancer, 18 upper urinary tract urothelial carcinoma [UTUC], 10 penile cancer and 33 testicular cancer). In comparison to the same period in 2019, 515 uro‐oncological surgical procedures were performed (313 prostate cancer, 45 bladder cancer, 98 renal cancer, 20 UTUC, 11 penile cancer, 26 testicular cancer and 2 adrenal cancer). There was an increase of 4% in the total number of surgical procedures from 2019 to 2020. The main increase was observed for renal, bladder and testicular cancer surgery with a 16% (*n* = 98 vs. 114), 24% (*n* = 45 vs. 56) and 27% increase (*n* = 26 vs. 33), respectively. On the other hand, UTUC surgery (i.e., nephroureterectomy) saw a 10% decline (*n* = 20 vs. 18), in addition to a 9% in penile (*n* = 11 vs. 10), and 100% decline in adrenal (*n* = 2 vs. 0) cancer surgery. Clinical and demographic characteristics for overall urological cancers and by cancer type are summarised in Tables 1 and S1. Age, sex, SES, ethnicity and comorbidities were all comparable between both periods (i.e., 90% male, 60% high SES and 37% with hypertension). Data on performance status were not available. Surgical outcomes of IEO patients are summarised in Table 3. When reviewing for ASA grade, 43 (9%) patients were ASA Grade III or higher (14 [5%] prostate cancer, 12 [21%] bladder cancer, 3 [17%] UTUC and 13 [11%] renal cancer). The median operative time was 210.5 min for all cancers, while the median theatre time was 273.5 min. Readmissions were required for less than 1% (1 [<1%] for prostate cancer and 1 [<1%] for renal cancer). ICU stay >24 h was required for one (<1%) bladder cancer patient, and two (3%) bladder cancer patients developed pneumonia in the postoperative period. As for postoperative COVID‐19 outcomes, only one bladder cancer patient developed COVID‐19, reporting mild/moderate disease. No patients died in the observed period. Tables 2 and 3 summarise surgical and COVID‐19 outcomes in IEO patients.

**TABLE 1:** Patient characteristics of European Institute of Oncology (IEO) and South East London Cancer Alliance (SELCA) urological cancer patients receiving radical surgery in 2019 and 2020

	IEO			SELCA		
	2019 (*n* = 515, %)	2020 (*n* = 534, %)	*p* value	2019 (*n* = 403, %)	2020 (*n* = 312, %)	*p* value
Difference (%)	+4%			−22.6%		
Sex						
Male	468 (90)	488 (91)	0.96	333 (83)	246 (80)	0.10
Female	47 (10)	46 (9)	0.96	70 (17)	66 (20)	0.10
Age						
<50	47 (9)	50 (9)	0.91	56 (14)	50 (16)	0.52
50–59	117 (23)	127 (24)	0.74	48 (12)	83 (27)	0.00
60–69	206 (40)	214 (40)	0.98	100 (25)	87 (28)	0.33
70–79	133 (26)	131 (25)	0.82	110 (28)	64 (20)	0.02
≥80	12 (2)	12 (2)	0.94	86 (22)	29 (9)	0.00
Mean (*SD*)	63 (10.98)	63 (10.41)	0.41	63 (14.7)	63 (14.3)	0.12
SES						
Low	41 (8)	26 (5)	0.11	48 (12)	58 (19)	0.01
Medium	118 (23)	105 (20)	0.11	178 (45)	145 (47)	0.43
High	298 (58)	323 (60)	0.32	151 (38)	101 (32)	0.13
Missing	58 (11)	80 (15)	0.10	23 (6)	4 (1)	0.00
Ethnicity						
White British	0	0		79 (20)	74 (2)	0.17
White other	513 (99)	532 (99)	0.54	24 (6)	27 (9)	0.13
Black Caribbean	0	0		9 (2)	6 (2)	0.76
Black African	1 (<1)	1 (<1)	0.31	10 (3)	8 (3)	0.95
Black other	1 (<1)	1 (<1)	0.98	17 (4)	9 (3)	0.32
Asian	0	0		5 (1)	0	0.02
Mixed	0	0		1 (<1)	2 (<1)	0.44
Other	0	0		4 (1)	1 (<1)	0.25
Unknown	0	0		251 (63)	181 (59)	0.26
Comorbidities						
Hypertension	185 (36)	189 (35)	0.52	115 (29)	8 (3)	0.00
Diabetes mellitus	40 (8)	37 (7)	0.61	46 (12)	21 (7)	0.02
Lung conditions	24 (5)	18 (3)	0.55	9 (2)	14 (4)	0.10
Renal impairment	8 (2)	12 (2)	0.37	67 (17)	3 (1)	0.00
Liver conditions	11 (2)	15 (3)	0.73	6 (2)	0	0.01
CVD	83 (16)	78 (15)	0.36	13 (3)	21 (7)	0.03
Performance status						
0	0	0		85 (21)	146 (47)	0.00
1	0	0		69 (17)	46 (15)	0.36
2	0	0		21 (5)	13 (4)	0.49
3	0	0		1 (<1)	2 (<1)	0.44
4	0	0		0	0	
Unknown	515 (100)	534 (100)		224 (56)	105 (34)	0.00

Abbreviations: CVD, cardiovascular disease; IEO, European Institute of Oncology; SELCA, South East London Cancer Alliance; SES, socioeconomic status.

**TABLE 2:** Surgical outcomes of IEO and SELCA urological cancer patients receiving radical treatment during COVID‐19 in 2020

	Prostate	Bladder	Kidney	UTUC	Penile	Testicular	Adrenal	Total
IEO								
	*n* = 303	*n* = 56	*n* = 114	*n* = 18	*n* = 10	*n* = 33	*n* = 0	*n* = 534
ASA Grade III/IV/V	14 (5)	12 (21)	13 (11)	3 (17)	1 (10)	0	0	43 (9)
Surgery time—min (median, IQR)	238,5 (114–387)	210,5 (155–525)	149 (70–328)	246,5 (144–397)	63 (52–129)	62.5 (47–124)	0	210,5 (51–429)
Theatre time—min (median, IQR)	303 (156–460)	310 (258–639)	244 (121–444)	329 (237–458)	115 (94–205)	112 (92–194)	0	273,5 (93–459)
ICU stay >24 h	0	1 (<1)	0	0	0	0	0	1(<1)
Pneumonia	0	2 (3)	0	0	0	0	0	2 (<1)
LOS—days	3	11	4	5	1	1	0	3
Readmissions	1 (<1)	0	1 (<1)	0	3	0	0	5 (1)
SELCA								
	*n* = 91	*n* = 117	*n* = 66	*n* = 6	*n* = 0	*n* = 24	*n* = 8	*n* = 312
ASA Grade III/IV/V	3 (3)	25 (21)	14 (21)	1 (17)	0	2 (8)	3 (38)	48 (15)
Surgery time—min (median, IQR)	173 (152–193)	44 (27–97)	145 (124–201)	150 (71–177)	0	60 (47–191)	161 (92–292)	145 (55–190)
Theatre time—min (median, IQR)	230 (201–251)	88 (67–160)	213 (182–273)	212 (127–239)	0	127 (96–321)	211 (154–371)	201 (113–254)
ICU stay >24 h	0	20 (17)	16 (24)	1 (17)	0	2 (8)	3 (38)	42 (13)
Pneumonia	0	0	0	0	0	0	0	
LOS—days	2	2	4	5	0	1	5	2
Re‐admissions	0	2 (2)	0	0	0	0	0	2 (<1%)

Abbreviations: ASA, American Society of Anaesthesiologists; ICU, intensive care unit; IEO, European Institute of Oncology; IQR, interquartile range; LOS, length of stay; SELCA, South East London Cancer Alliance; SES, socioeconomic status; UTUC, upper urinary tract urothelial carcinoma.

**TABLE 3:** COVID outcomes of surgical cancer patients receiving radical surgery during COVID‐19 in 2020

	Prostate	Bladder	Kidney	UTUC	Penile	Testicular	Adrenal	Total
IEO								
	*n* = 303	*n* = 56	*n* = 114	*n* = 18	*n* = 10	*n* = 33	*n* = 0	*n* = 534
COVID status								
Negative	303 (100)	55 (98)	114 (100)	18 (100)	10	33	0	490 (99)
Positive	0	1 (2)	0	0	0	0	0	1 (<1)
COVID severity								
Mild and moderate	0	1 (2)	0	0	0	0	0	1 (<1)
Severe	0	0	0	0	0	0	0	0
Death								
All‐cause (30 days)	0	0	0	0	0	0	0	0
All‐cause (90 days)	0	0	0	0	0	0	0	0
COVID‐19	0	0	0	0	0	0	0	0
SELCA								
	*n* = 91	*n* = 117	*n* = 66	*n* = 6	*n* = 0	*n* = 24	*n* = 8	*n* = 312
COVID status								
Positive	0	1 (<1)	0	0	0	0	0	1 (<1)
Death								
All‐cause (30 days)	0	2 (1)	0	0	0	0	0	2 (<1)
All‐cause (90 days)	1 (1)	4 (3)	0	0	0	0	0	5 (1)
COVID‐19	0	0	0	0	0	0	0	0

Abbreviations: IEO, European Institute of Oncology; SELCA, South East London Cancer Alliance; UTUC, upper urinary tract urothelial carcinoma.

**FIGURE 2:**
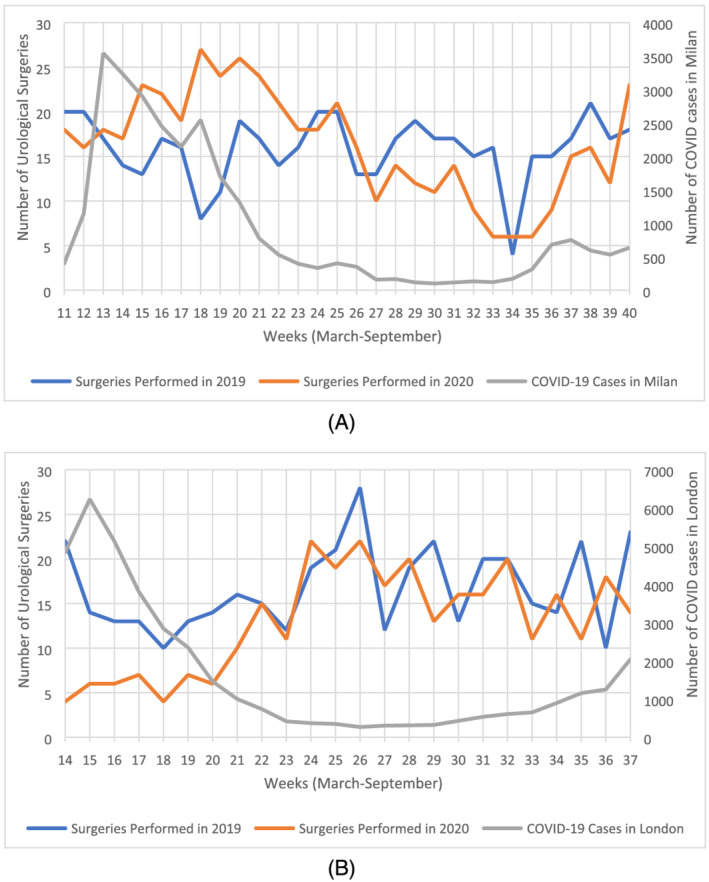
(A) Chart illustrating weekly COVID‐19 cases in Milan and number of surgeries performed in European Institute of Oncology (IEO) between 01/03/19 and 30/09/19 and 01/03/20 and 30/09/20. (B) Chart illustrating weekly COVID‐19 cases in London and number of surgeries performed in South East London Cancer Alliance (SELCA) between 23/03/19 and 08/09/19 and 23/03/20 and 08/09/20

Figure 2A illustrates the number of weekly COVID‐19 cases in Milan and uro‐oncological surgical procedures performed per week for 1 March to 30 September 2020, as well as the comparable period in 2019. There was a significant increase in the number of procedures performed in the first 12 weeks observed. Between Weeks 23 and 28 (31 June–5 July), the number of procedures was similar to the comparable period in 2019. From Week 28 onwards, the number of procedures in 2020 was consistently lower than the comparable period in 2019. The number of COVID‐19 cases in the Milan region had a steep rise during the first 4 weeks observed, reaching 3545 cases in Week 13 (15–21 March). Subsequently, the number of weekly COVID‐19 cases began to gradually decrease with the exception of a second smaller peak of 2546 cases in Week 18 (20–26 March).

At SELCA, 312 radical uro‐oncological surgical procedures were performed from 23 March to 8 September 2020 (91 prostate cancer, 117 bladder cancer, 66 renal cancer, 6 UTUC, 24 testicular cancer and 8 adrenal cancer). There was a decline of 23% in the number of surgical procedures compared to the same period in 2019 (156 prostate cancer, 121 bladder cancer, 82 renal cancer, 4 UTUC, 4 penile cancer, 35 testicular cancer and 1 adrenal cancers). The most notable decline was seen for prostate (42%, *n* = 91 vs. 156), testicular (46%, *n* = 24 vs. 35) and penile (100%, *n* = 0 vs. 4) cancer surgery. However, there was an increase in the number of UTUC (50%, *n* = 6 vs. 4) and adrenal (700%, *n* = 8 vs. 1) cancer surgery. Clinical and demographic characteristics are shown in Tables 1 and S2. The different patient characteristics when comparing 2020 with 2019 (i.e., 61% vs. 82% male, 29% vs. 50% aged >70 years, 3% vs. 29% with hypertension) likely reflects the clinical decision making for COVID‐19 vulnerable patients.

Surgical outcomes for SELCA patients are summarised in Table 3. Forty‐three patients (15%) had an ASA grade III or higher (3 [3%] prostate cancer, 25 [21%] bladder cancer, 14 [21%] renal cancer, 1 [17%] UTUC, 2 [8%] testicular cancer, 3 [38%] adrenal cancer). Median operative time was 145 min and median theatre time was 201 min for all cancers combined. ICU stay >24 h was required for 41 (13%) of patients (20 [17%] bladder cancer, 16 [24%] renal cancer, 1 [17%] UTUC, 2 [8%] testicular cancer and 3 [38%] adrenal cancer). No patients developed pneumonia postoperatively. Readmissions were required for 2 (2%) bladder cancer patients. Of the total SELCA patients undergoing uro‐oncological surgery, only one bladder cancer patient developed COVID‐19. There was no COVID‐19 related mortality. Data were not available on COVID‐19 severity. Two (1%) bladder cancer patients died of any cause within 30 days of surgery. Five (1%) of all uro‐oncological cancer patients died within 90 days of surgery. COVID‐19 outcomes for cancer patients undergoing radical surgery are summarised in Table 3.

Figure 2B illustrates the number of weekly COVID‐19 cases in London and uro‐oncological surgical procedures performed per week for 23 March to 8 September 2020, as well as the comparable period in 2019. There was a significant decrease in the number of procedures throughout the first 6 weeks of the observed period (23 March 23 to 10 May). In Week 20, the number of procedures began to gradually increase in 2020 and by Week 22 the number of procedures performed in 2020 were equal to the comparable period in 2019. From Week 24 onwards, uro‐oncological surgical procedures performed in 2020 varied in number and had similar peaks to 2019. On the other hand, COVID‐19 cases began to decline from Week 17 onwards and maintained a plateau until cases began to rise again starting Week 30 (13 July).

## 4 DISCUSSION

Milan and London were both at the epicentre of the first COVID‐19 wave. Although an increase in the number of uro‐oncological surgical procedures was observed in Milan, there was a decrease in the number of surgical procedures in London. The latter was likely due to clinical prioritisation across all time critical cancer surgery by NHS England. Moreover, it should be noted that SELCA hospitals were considered COVID‐19 hubs and thus, faced reductions in hospital beds and scheduled surgical procedures. This was due to increased COVID‐19 hospitalisations and redeployment of anaesthesiologists for the airway management of COVID‐19 patients. However, the implemented COVID‐19 minimal ‘green’ pathways were shown to be safe for all urological cancer patients requiring radical treatment, with limited complications and almost no peri‐operative COVID‐19 infections in both groups and no COVID‐19‐related mortality. These findings are consistent with our previous study looking at overall surgical practice in both cancer hubs which found that although a decline in the number of surgical procedures was observed, the implemented COVID‐19 pathways were shown to be safe for cancer patients.^10^

Regardless of the high number of COVID‐19 cases reported in the first wave of the pandemic in the metropolitan area of Milan, the IEO saw a 3.7% increase in the number of uro‐oncological surgical procedures performed. Similar results were reported by Ingels et al. who described a limited impact on perioperative complications in eight urological centres in Paris where oncological surgery was prioritised during the first 4 weeks of the pandemic.^20^ However, two other observational studies reported a decline in the number overall urological surgical procedures (including uro‐oncology surgery) during the COVID‐19 pandemic.^8^, ^21^ The IEO was not a COVID‐19 hub and thus was chosen by the region as a reference centre for other non‐operative hospitals for pandemic urgency. Several urological cancer patients were sent from these hospitals to the IEO, which may explain the increase in number of uro‐oncological procedures.^10^ Moreover, surgery for bladder (24%, *n* = 45 vs. 56), renal (16%, *n* = 98 vs. 114) and testicular (27%, *n* = 26 vs. 33) cancer saw the largest increase in volume in the IEO population. This was in line with various other studies, which suggested that radical cystectomy (RC) should be prioritised, as delays exceeding 90 days between diagnosis and RC are associated with decreased overall survival.^22^, ^23^ This was also supported with a survey from 28 Italian urology centres comparing surgical procedures performed in 2020 to 2019 that reported an increase in the number of RC procedures performed in 2020.^24^ Similarly, advanced renal cancers require a higher priority for timely surgical care.^25^, ^26^ A recent systematic review by Tachibana et al. came to the conclusion that high‐stage renal cancer should always be considered for early surgery, while low‐stage renal cancer can be delayed until adequate resources are available.^27^ There is limited evidence looking at testicular cancer surgery during the COVID‐19 pandemic; however, a review of the current literature suggests that new testicular cancer diagnoses should receive priority care, including surgical treatment (^27^, REF mayor). On the other hand, UTUC surgery saw a 10% decline in 2020 compared with 2019. This is not in line with previous studies that have found that delay in surgical time likely affects overall survival outcomes in high risk cases.^27^ However, it is should be noted that the 10% decline is attributed to a difference of only two patients between the two periods (20 vs. 18). Thus, this decline may is not clinically significant and may be explained by a casual variability.

In our SELCA population, overall urological surgery had a decline of 26% (n = 403 vs. 308). The largest decline was seen for prostate (−42%, n = 156 vs. 91), penile (−100%, n = 4 vs. 0) and testicular (−31%, n = 35 vs. 24) cancer. The decrease in the number of prostate cancer procedures is in line with various studies that reported delays in up to 12 months did not have worse oncologic outcomes in low/moderate risk patients.^28^, ^29^ In addition, it has also been reported that neoadjuvant hormonal therapy does not negatively impact long‐term survival and allows patients to safely delay surgery.^27^ Moreover, the decline in the number of penile cancer surgery is also in line with published studies. It has been reported that topical treatment is effective and should be the first option in the absence of lymph node involvement, while radiotherapy has had good results in more advanced lesions.^30^ As previously mentioned for testicular cancer, there is limited information on the effects of delaying surgical treatment due to COVID‐19. However, a recent review reported that testicular cancer patients would benefit from minimised delays and their treatment should be prioritised.^27^ There was a 300% increase in the number of adrenal surgeries (*n* = 1 vs. 4); this is likely due to the associated endocrine abnormalities from hormone secreting tumours and the need for early surgical treatment.^31^, ^32^

In both hubs, the decision making for the surgical prioritisation was individually reviewed by a virtual tumour board and treatment plans were personalised taking into account the patients' clinical characteristics. In the SELCA population, some patient characteristics were fairly different when comparing 2020 with 2019 (i.e., 61% vs. 82% male, 29% vs. 50% aged >70 years, 3% vs. 29% with hypertension), which likely reflects the clinical decision making for COVID‐19 vulnerable patients. The risks and benefits of each procedure should be assiduously weighed against the potential risk of contracting COVID‐19.^10^ The deferral of cancer treatment has created a backlog of patients and this will likely have great implications for both patients and healthcare workers. Although we are aware that early reports suggest patients are at high risk of perioperative infection and subsequent high risk of mortality, there is increasing epidemiological evidence suggesting that the risk of COVID‐19 infection is minimal with safety precautions.^9^, ^20^, ^27^ Thus, it is critical for more cancer centres to begin to implement COVID‐19 pathways and reintroduce elective cancer surgery to prevent more delays in oncological care.

The current study is among the first large observational study looking at safe pathways for uro‐oncological surgical procedures implemented in two cancer hubs, in Milan and South‐East London. Further studies are needed stratifying cancer subtypes and stages, as well as the types of surgical procedures performed to carry out an in‐depth review into the safest pathways for cancer patients. In addition, a more detailed description of the criteria used for prioritisation of cancer surgery is needed to further inform future clinical guidelines. The major limitations of our study include the lack of data on: COVID‐19 severity for SELCA patients, COVID‐19 test results of healthcare staff in both centres, and COVID‐19 test results in the postoperative period. Moreover, the differences in each cancer hub's COVID‐19 ‘green’ pathway may be a potential source for bias.

## 5 CONCLUSIONS

The findings from our study suggest that the COVID‐19 minimal ‘green’ pathways implemented in our study populations are safe for patients who require radical treatment for genitourinary malignancy. Continuation of major surgery for urological cancer should be encouraged during the ongoing COVID‐19 pandemic provided appropriately designed preventative pathways to avoid the spread of COVID‐19 are implemented. Moreover, it is critical for all urological cancer centres to identify measures to manage the backlog of cancer patients awaiting treatment either through initial deferral or delayed referral.

## DISCLOSURE OF INTERESTS

None.

## Supporting information


**Table S1.** Patient characteristics of IEO urological cancer patients receiving radical surgery between in 2019 and 2020, divided by cancer type.
**Table S2.** Patient characteristics of SELCA urological cancer patients receiving radical surgery between in 2019 and 2020, divided by cancer type.
