# Supplementary material for: Impact of the COVID‐19 pandemic on urological cancers: The surgical experience of two cancer hubs in London and Milan
Source: BJUI Compass. 2022 Jan 27;3(4):277–86. doi: 10.1002/bco2.135 (PMC9231679; doi:10.1002/bco2.135)
Supplement: Supplementary file 1 — Table S1. Patient characteristics of IEO urological cancer patients receiving radical surgery between in 2019 and 2020, divided by cancer type. Table S2. Patient characteristics of SELCA urological cancer patients receiving radical surgery between in 2019 and 2020, divided by cancer type. [file BCO2-3-277-s001.docx]

**Supplementary table 1.** Patient characteristics of IEO urological cancer patients receiving radical surgery between in 2019 and 2020, divided by cancer type.

|  | **Prostate** | |  | **Bladder** | |  | **Kidney** | |  | **UTUC** | |  |
| --- | --- | --- | --- | --- | --- | --- | --- | --- | --- | --- | --- | --- |
|  | **2019**  **(n=313)** | **2020**  **(n=303)** | **P-value** | **2019**  **(n=45)** | **2020**  **(n=56)** | **P-value** | **2019**  **(n=98)** | **2020**  **(n=114)** | **P-value** | **2019**  **(n=20)** | **2020**  **(n=18)** | **P-value** |
| **Difference (%)** | **-3%** | |  | **+24%** | |  | **+16%** | |  | **-10%** | |  |
| **Sex** | | | | | | | | | | | | |
| Male | 313 (100) | 303 (100) |  | 37 | 46 | 0.99 | 67 | 84 | 0.39 | 14 | 13 | 0.87 |
| Female | 0 | 0 |  | 8 | 10 | 0.99 | 31 | 30 | 0.39 | 6 | 5 | 0.87 |
| **Age** | | | | | | | | | | | | |
| <50 | 5 (2) | 7 (2) | 0.52 | 3 | 1 | 0.23 | 16 | 15 | 0.51 | 0 | 1 | 0.30 |
| 50-59 | 77 (25) | 69 (23) | 0.59 | 4 | 12 | 0.07 | 30 | 41 | 0.40 | 3 | 0 | 0.06 |
| 60-69 | 145 (46) | 151 (50) | 0.38 | 18 | 18 | 0.41 | 30 | 33 | 0.79 | 8 | 5 | 0.42 |
| 70-79 | 86 (27) | 76 (25) | 0.49 | 16 | 20 | 0.98 | 20 | 24 | 0.90 | 6 | 9 | 0.20 |
| ≥80 | 0 (0) | 0 |  | 4 | 5 | 0.99 | 2 | 1 | 0.48 | 3 | 3 | 0.88 |
| Mean (SD) | 63.9 (6.8) | 64 (8.6) |  | 67.8 (10) | 64.1 (8.6) |  | 67.8 (8.6) | 64.1 (8.6) |  | 68.6 (8.7) | 64.3 (8.5) |  |
| **SES** | | | | | | | | | | | | |
| Low | 17 (5) | 13 (4) | 0.50 | 5 (11) | 5 (9) | 0.71 | 10 (10) | 5 (4) | 0.10 | 4 (20) | 2 (11) | 0.44 |
| Medium | 76 (24) | 61 (20) | 0.21 | 14 (31) | 13 (23) | 0.37 | 19 (20) | 16 (15) | 0.29 | 4 (20) | 6 (33) | 0.34 |
| High | 181 (58) | 192 (63) | 0.15 | 23 (51) | 26 (46) | 0.63 | 59 (60) | 72 (63) | 0.65 | 10 (50) | 7 (39) | 0.48 |
| Missing | 39 (12) | 37 (12) | 0.92 | 3 (7) | 12 (21) | 0.02 | 10 (10) | 21 (18) | 0.08 | 2 (10) | 3 (17) | 0.54 |
| **Ethnicity** | | | | | | | | | | | | |
| White Other | 313 (100) | 303 (100) |  | 45 (100) | 56 (100) |  | 96 (96) | 113 (99) | 0.48 | 20 (100) | 18 (100) |  |
| Black African | 0 | 0 |  | 0 | 0 |  | 1 (2) | 0 | 0.31 | 0 | 0 |  |
| Black Other | 0 | 0 |  | 0 | 0 |  | 1 (2) | 1 (1) | 0.91 | 0 | 0 |  |
| **Comorbidities** | | | | | | | | | | | | |
| Hypertension | 112 (36) | 103 (34) | 0.64 | 22 (49) | 28 (50) | 0.91 | 36 (37) | 42 (37) | 0.98 | 14 (70) | 7 (39) | 0.04 |
| Diabetes Mellitus | 24 (8) | 14 (5) | 0.11 | 7 (16) | 10 (18) | 0.75 | 7 (7) | 9 (8) | 0.83 | 1 (5) | 3 (17) | 0.24 |
| Lung Conditions | 7 (2) | 8 (3) | 0.74 | 8 (18) | 8 (14) | 0.63 | 3 (3) | 0 | 0.07 | 3 (15) | 2 (11) | 0.72 |
| Renal Impairment | 3 (1) | 4 (1) | 0.67 | 2 (4) | 4 (7) | 0.55 | 2 (2) | 3 (3) | 0.77 | 0 | 0 |  |
| Liver Conditions | 6 (2) | 6 (2) | 0.95 | 0 | 3 (5) | 0.07 | 4 (4) | 1 (1) | 0.14 | 1 (5) | 2 (11) | 0.49 |
| CVD | 46 (15) | 44 (15) | 0.47 | 14 (31) | 12 (21) | 0.27 | 17 (17) | 14 (12) | 0.30 | 5 (25) | 4 (22) | 0.84 |
| **Performance status** | | | | | | | | | | | | |
| Unknown | 313 (100) | 303 (100) |  | 45 (100) | 56 (100) |  | 98 (100) | 114 (100) |  | 20 (100) | 18 (100) |  |

**Supplementary Table 1 (continuation).** Patient characteristics of IEO urological cancer patients receiving radical surgery between in 2019 and 2020, divided by cancer type.

|  | **Penile** | |  | **Testicular** | |  | **Adrenal** | |
| --- | --- | --- | --- | --- | --- | --- | --- | --- |
|  | **2019**  **(n=11)** | **2020**  **(n=10)** | **P-value** | **2019**  **(n=26)** | **2020**  **(n=33)** | **P-value** | **2019**  **(n=2)** | **2020**  **(n=0)** |
| **Difference (%)** | **-9%** | |  | **27%** | |  | **-100%** | |
| **Sex** | | | | | | | | |
| Male | 11 | 10 |  | 26 | 33 |  | 0 | 0 |
| Female | 0 | 0 |  | 0 | 0 |  | 2 | 0 |
| **Age** | | | | | | | | |
| <50 | 1 | 2 | 0.47 | 22 | 24 | 0.25 | 0 | 0 |
| 50-59 | 1 | 1 | 0.94 | 1 | 4 | 0.22 | 1 | 0 |
| 60-69 | 4 | 5 | 0.52 | 1 | 2 | 0.69 | 0 | 0 |
| 70-79 | 4 | 1 | 0.12 | 1 | 1 | 0.86 | 0 | 0 |
| ≥80 | 1 | 1 | 0.94 | 1 | 2 | 0.69 | 1 | 0 |
| Mean (SD) | 69 (12.64) | 60 (13.66) |  | 38 (15.65) | 45 (16) |  | 67 (18,38) | 0 |
| **Socioeconomic status** | | | | | | | | |
| Low | 4 | 1 | 0.12 | 0 | 0 |  | 1 | 0 |
| Medium | 3 | 3 | 0.89 | 2 | 6 | 0.21 | 0 | 0 |
| High | 4 | 2 | 0.39 | 20 | 24 | 0.71 | 1 | 0 |
| Missing | 0 | 4 | 0.00 | 4 | 3 | 0.46 | 0 | 0 |
| **Ethnicity** | | | | | | | | |
| White Other | 11 | 10 |  | 26 | 32 | 0.30 | 2 | 0 |
| Black African | 0 | 0 |  | 0 | 1 |  | 0 | 0 |
| **Comorbidities** | | | | | | | | |
| Hypertension | 3 | 5 | 0.27 | 0 | 4 | 0.03 | 1 | 0 |
| Diabetes Mellitus | 0 | 1 | 0.29 | 0 | 0 |  | 1 | 0 |
| Lung Conditions | 2 | 0 | 0.11 | 1 | 0 | 0.30 | 0 | 0 |
| Renal Impairment | 0 | 0 |  | 1 | 1 | 0.86 | 0 | 0 |
| Liver Conditions | 0 | 2 | 0.11 | 0 | 0 |  | 0 | 0 |
| CVD | 0 | 0 |  | 1 | 4 | 0.22 | 0 | 0 |
| **Performance status** | | | | | | | | |
| Unknown | 11 | 10 |  | 26 | 33 | 0.16 | 2 | 0 |

**Supplementary Table 2.** Patient characteristics of SELCA urological cancer patients receiving radical surgery between in 2019 and 2020, divided by cancer type.

|  | **Prostate** | |  | **Bladder** | |  | **Kidney** | |  | **UTUC** | |  |
| --- | --- | --- | --- | --- | --- | --- | --- | --- | --- | --- | --- | --- |
|  | **2019**  **(n=156)** | **2020**  **(n=91)** | **P-value** | **2019**  **(n=121)** | **2020**  **(n=117)** | **P-value** | **2019**  **(n=82)** | **2020**  **(n=66)** | **P-value** | **2019**  **(n=4)** | **2020**  **(n=6)** | **P-value** |
| **Difference (%)** | **-42%** | |  | **-3%** | |  | **-20%** | |  | **+50** | |  |
| **Sex** | | | | | | | | | | | | |
| Male | 156 (100) | 91 (100) |  | 81 (67) | 86 (74) | 0.26 | 53 (65) | 33 (50) | 0.07 | 3 (75) | 4 (67) | 0.77 |
| Female | 0 | 0 |  | 40 (33) | 31 (26) | 0.26 | 29 (35) | 33 (50) | 0.07 | 1 (25) | 2 (33) | 0.77 |
| **Age** | | | | | | | | | | | | |
| <50 | 11 (7) | 5 (5) | 0.62 | 6  (5) | 10 (9) | 0.26 | 8 (10) | 15 (23) | 0.03 | 0 | 1 (17) | 0.27 |
| 50-59 | 65 (42) | 39 (43) | 0.85 | 11 (9) | 18 (15) | 0.13 | 21 (26) | 19 (29) | 0.66 | 1 (25) | 1 (17) | 0.75 |
| 60-69 | 60 (38) | 38 (42) | 0.61 | 20 (17) | 25 (21) | 0.34 | 25 (30) | 21 (32) | 0.86 | 1 (25) | 0 | 0.24 |
| 70-79 | 17 (11) | 9 (10) | 0.80 | 42 (35) | 41 (35) | 0.95 | 25 (30) | 7 (11) | 0.00 | 2 (50) | 3 (50) | 1 |
| ≥80 | 3 (2) | 0 | 0.08 | 42 (35) | 23 (20) | 0.00 | 3 (4) | 4 (6) | 0.50 | 0 | 1 (17) | 0.27 |
| Mean (SD) | 60 (8.2) | 60 (7.4) |  | 76 (12.7) | 71 (12.8) |  | 65 (10.8) | 59 (13.6) |  | 71 (10.5) | 71 (14.4) |  |
| **SES** | | | | | | | | | | | | |
| Low | 20 (13) | 15  (16) | 0.43 | 15 (12) | 23 (20) | 0.12 | 8 (10) | 13 (20) | 0.09 | 0 | 2 (33) | 0.08 |
| Medium | 65 (42) | 45 (49) | 0.23 | 55 (45) | 61 (52) | 0.30 | 38 (46) | 26 (39) | 0.39 | 0 | 1 (17) | 0.27 |
| High | 65 (42) | 31 (34) | 0.23 | 42 (35) | 30 (26) | 0.12 | 28 (34) | 26 (39) | 0.51 | 4 (100) | 3 (50) | 0.01 |
| Missing | 6 (4) | 0 | 0.01 | 9 (7) | 3 (3) | 0.08 | 8 (10) | 1 (2) | 0.02 | 0 | 0 |  |
| **Ethnicity** | | | | | | | | | | | | |
| White British | 20 (13) | 13 (14) | 0.74 | 40 (33) | 34 (29) | 0.50 | 13 (16) | 14 (21) | 0.40 | 1 (25) | 3 (50) | 0.40 |
| White Other | 9 (6) | 3 (3) | 0.34 | 6 (5) | 13 (11) | 0.07 | 5 (6) | 9 (14) | 0.13 | 0 | 1 (17) | 0.27 |
| Black Caribbean | 7 (4) | 2 (2) | 0.31 | 0 | 3 (3) | 0.07 | 1 (1) | 0 | 0.31 | 0 | 1 (17) | 0.27 |
| Black African | 6 (4) | 3 (3) | 0.82 | 3 (2) | 3 (3) | 0.96 | 0 | 2 (3) | 0.15 | 0 | 0 |  |
| Black Other | 7 (4) | 8 (9) | 0.20 | 4 (3) | 0 | 0.04 | 5 (6) | 0 | 0.02 | 0 | 0 |  |
| Asian | 2 (1) |  | 0.15 | 1 (1) | 0 | 0.31 | 1 (1) | 0 | 0.31 | 1 (25) |  | 0.24 |
| Mixed | 1 (1) | 1  (1) | 0.71 | 0 | 0 |  | 0 | 1 (2) | 0.31 | 0 | 0 |  |
| Other | 2 (1) | 1  (1) | 0.89 | 0 | 0 |  | 1 (1) | 0 | 0.31 | 1 (25) | 0 | 0.24 |
| Unknown | 102 (65) | 60  (66) | 0.93 | 67 (55) | 64 (55) | 0.91 | 56 (68) | 40 (61) | 0.33 | 1 (25) | 1 (17) | 0.75 |
| **Comorbidities** | | | | | | | | | | | | |
| Hypertension | 35 (22) | 0 | 0.00 | 44 (36) | 5 (4) | 0.00 | 30 (37) | 3 (5) | 0.00 | 3 (75) | 0 | 0.00 |
| Diabetes Mellitus | 12 (8) | 1  (1) | 0.00 | 22 (18) | 13 (1) | 0.12 | 11 (13) | 5 (8) | 0.24 | 1 (25) | 0 | 0.24 |
| Lung Conditions | 0 | 0 |  | 3 (2) | 10 (9) | 0.03 | 6 (7) | 3 (5) | 0.47 | 0 | 0 |  |
| Renal Impairment | 0 | 0 |  | 0 | 3 (3) | 0.07 | 66 (80) | 0 | 0.00 | 1 (25) | 0 | 0.24 |
| Liver Conditions | 1 (1) | 0 | 0.31 | 4 (3) | 0 | 0.04 | 1 (1) | 0 | 0.31 | 0 | 0 |  |
| CVD | 6 (4) | 1  (1) | 0.14 | 5 (4) | 14 (12) | 0.02 | 2 (2) | 4 (6) | 0.28 | 0 | 0 |  |
| **Performance status** | | | | | | | | | | | | |
| 0 | 67 (43) | 67 (74) | 0.00 | 6  (5) | 37 (32) | 0.00 | 6 (7) | 28 (42) | 0.00 | 0 | 1  (17) | 0.27 |
| 1 | 24 (15) | 8 (9) | 0.11 | 30 (25) | 21 (18) | 0.19 | 9 (11) | 9 (14) | 0.62 | 0 | 2  (33) | 0.08 |
| 2 | 12 (8) | 2 (2) | 0.03 | 7 (6) | 8 (7) | 0.73 | 2 (2) | 1 (2) | 0.68 | 0 | 0 |  |
| 3 | 1 (1) | 1 (1) | 0.71 | 0 | 1 (1) | 0.31 | 0 | 0 |  | 0 | 0 |  |
| 4 | 0 | 0 |  | 0 | 0 |  | 0 | 0 |  | 0 | 0 |  |
| Unknown | 52 (33) | 13  (14) | 0.00 | 78 (64) | 50 (43) | 0.00 | 65 (79) | 28 (42) | 0.00 | 4 (100) | 3 (50) | 0.01 |

**Supplementary Table 2 (continuation).** Patient characteristics of SELCA urological cancer patients receiving radical surgery between in 2019 and 2020, divided by cancer type.

|  | **Penile** | | **Testicular** | |  | **Adrenal** | |  |
| --- | --- | --- | --- | --- | --- | --- | --- | --- |
|  | **2019**  **(n=4)** | **2020**  **(n=0)** | **2019**  **(n=35)** | **2020**  **(n=24)** | **P-value** | **2019**  **(n=1)** | **2020**  **(n=8)** | **P-value** |
| **Difference (%)** | **-100%** | | **-46%** | |  | **+700%** | |  |
| **Sex** | | | | | | | | |
| Male | 2 | 0 | 35 | 24 |  | 1 | 4 | 0.00 |
| Female | 0 | 0 | 0 | 0 |  | 0 | 4 |  |
| <50 | 0 | 0 | 31 | 17 | 0.09 | 0 | 2 | 0.10 |
| 50-59 | 0 | 0 | 2 | 5 | 0.09 | 0 | 1 | 0.28 |
| 60-69 | 2 | 0 | 2 | 2 | 0.70 | 1 | 2 | 0.00 |
| 70-79 | 0 | 0 | 0 | 0 |  | 0 | 3 | 0.02 |
| ≥80 | 0 | 0 | 0 | 0 |  | 0 | 0 |  |
| Mean (SD) | 65 (1.4) |  | 31 (11.3) | 35 (16) |  | 60 | 62 (19.8) |  |
| **Socioeconomic status** | | | | | | | | |
| Low | 0 | 0 | 5 | 6 | 0.31 | 0 | 0 |  |
| Medium | 1 | 0 | 19 | 10 | 0.33 | 0 | 5 | 0.00 |
| High | 1 | 0 | 11 | 8 | 0.87 | 0 | 3 | 0.02 |
| Missing | 0 | 0 | 0 | 0 |  | 1 | 0 |  |
| **Ethnicity** | | | | | | | | |
| White British | 1 | 0 | 4 | 7 | 0.09 | 0 | 4 | 0.00 |
| White Other | 0 | 0 | 4 | 2 | 0.69 | 0 | 0 |  |
| Black Caribbean | 0 | 0 | 1 | 0 | 0.31 | 0 | 0 |  |
| Black African | 0 | 0 | 1 | 0 | 0.31 | 0 | 0 |  |
| Black Other | 0 | 0 | 1 | 0 | 0.31 | 0 | 1 | 0.28 |
| Asian | 0 | 0 | 0 | 0 |  | 0 | 0 |  |
| Mixed | 0 | 0 | 0 | 0 |  | 0 | 0 |  |
| Other | 0 | 0 | 0 | 0 |  | 0 | 0 |  |
| Unknown | 1 | 0 | 24 | 15 | 0.63 | 1 | 3 | 0.00 |
| **Comorbidities** | | | | | | | | |
| Hypertension | 0 | 0 | 3 | 0 | 0.07 | 0 | 0 |  |
| Diabetes Mellitus | 0 | 0 | 0 | 1 | 0.30 | 0 | 1 | 0.28 |
| Lung Conditions | 0 | 0 | 0 | 0 |  | 0 | 1 | 0.28 |
| Renal Impairment | 0 | 0 | 0 | 0 |  | 0 | 0 |  |
| Liver Conditions | 0 | 0 | 0 | 0 |  | 0 | 0 |  |
| CVD | 0 | 0 | 0 | 2 | 0.13 | 0 | 0 |  |
| **Performance status** | | | | | | | | |
| 0 | 0 | 0 | 6 | 11 | 0.01 | 0 | 2 | 0.10 |
| 1 | 0 | 0 | 6 | 3 | 0.61 | 0 | 3 | 0.02 |
| 2 | 0 | 0 | 0 | 2 | 0.13 | 0 | 0 |  |
| 3 | 0 | 0 | 0 | 0 |  | 0 | 0 |  |
| 4 | 0 | 0 | 0 | 0 |  | 0 | 0 |  |
| Unknown | 2 | 0 | 23 | 8 | 0.00 | 1 | 3 | 0.00 |
